# Heterogeneous Family of Cyclomodulins: Smart Weapons That Allow Bacteria to Hijack the Eukaryotic Cell Cycle and Promote Infections

**DOI:** 10.3389/fcimb.2017.00208

**Published:** 2017-05-23

**Authors:** Rachid A. El-Aouar Filho, Aurélie Nicolas, Thiago L. De Paula Castro, Martine Deplanche, Vasco A. De Carvalho Azevedo, Pierre L. Goossens, Frédéric Taieb, Gerard Lina, Yves Le Loir, Nadia Berkova

**Affiliations:** ^1^STLO, Agrocampus Ouest Rennes, Institut National de la Recherche AgronomiqueRennes, France; ^2^Departamento de Biologia Geral, Laboratório de Genética Celular e Molecular (LGCM), Instituto de Ciências Biológicas, Universidade Federal de Minas GeraisBelo Horizonte, Brazil; ^3^HistoPathologie et Modèles Animaux/Pathogénie des Toxi-Infections Bactériennes, Institut PasteurParis, France; ^4^CHU Purpan USC INRA 1360-CPTP, U1043 Institut National de la Santé et de la Recherche Médicale, Pathogénie Moléculaire et Cellulaire des Infections à Escherichia coliToulouse, France; ^5^International Center for Infectiology ResearchLyon, France; ^6^Centre National de la Recherche Scientifique, UMR5308, Institut National de la Santé et de la Recherche Médicale U1111, Ecole Normale Supérieure de Lyon, Université Lyon 1Lyon, France; ^7^Département de Biologie, Institut des Agents Infectieux, Hospices Civils de LyonLyon, France

**Keywords:** eukaryotic cell cycle alteration, bacterial toxins, cyclomodulins, colonization, invasion, infective efficiency, reduced host response

## Abstract

Some bacterial pathogens modulate signaling pathways of eukaryotic cells in order to subvert the host response for their own benefit, leading to successful colonization and invasion. Pathogenic bacteria produce multiple compounds that generate favorable conditions to their survival and growth during infection in eukaryotic hosts. Many bacterial toxins can alter the cell cycle progression of host cells, impairing essential cellular functions and impeding host cell division. This review summarizes current knowledge regarding cyclomodulins, a heterogeneous family of bacterial effectors that induce eukaryotic cell cycle alterations. We discuss the mechanisms of actions of cyclomodulins according to their biochemical properties, providing examples of various cyclomodulins such as cycle inhibiting factor, γ-glutamyltranspeptidase, cytolethal distending toxins, shiga toxin, subtilase toxin, anthrax toxin, cholera toxin, adenylate cyclase toxins, vacuolating cytotoxin, cytotoxic necrotizing factor, Panton-Valentine leukocidin, phenol soluble modulins, and mycolactone. Special attention is paid to the benefit provided by cyclomodulins to bacteria during colonization of the host.

## Bacterial pathogens hijack host defense response

Many pathogenic bacteria use sophisticated mechanisms to interfere with eukaryotic cells, to subvert the host response for their own benefit, and thus to colonize and invade the host tissues (Bhavsar et al., 2007; Alto and Orth, 2012). Bacterial pathogens can precisely target specific host cell activities such as cytoskeletal organization, cell cycle progression, vesicular trafficking and/or apoptosis using different effectors and delivery pathways (Zhou and Elledge, 2000). To do so, they produce substances, whose activity on the host cell results in an increased survival and replication and thus a better dissemination. These substances can manipulate pathways that regulate eukaryotic host cells (Alto and Orth, 2012). They can alter the host immune response, for example, by corrupting of MAP-signaling and NF-κB-pathway, major players of the immune response (Orth et al., 1999; Krachler et al., 2011; Lim and Staudt, 2013). Moreover, some bacterial virulence factors target evolutionarily conserved ubiquitylation machinery that regulates multiple cellular processes, including development, transcription, replication, cell signaling and immune function as shown in plant pathogens (e.g., *Pseudomonas syringae* pv. *tomato*) as well as animal or human pathogens (e.g., *Escherichia coli* or *Citrobacter rodentium*) (Huibregtse and Rohde, 2014). They can interact with eukaryotic modulating factors involved in the assembly of actin filaments, as was shown for the Rho GTPases-activating toxins such as the Cytotoxic Necrotizing Factor 1 described in *E. coli* (Bhavsar et al., 2007; Lemichez and Aktories, 2013). Their activity can ultimately hijack host response despite the negative pressure of the host immune system and induce a belated apoptosis of host cells bearing pathogens, which results in an extension of the time lapse for their replication.

To bypass the extracellular milieu and the membrane barrier, the bacterial effectors involved in such activities can be injected into the host eukaryotic cytoplasm, by specific injection systems such as Type III or Type IV Secretion Systems as demonstrated in Gram negative pathogens like enteric *Escherichia coli, Salmonella, Yersinia*, and *Shigella* (T3SS) or in *Brucella* sp. (T4SS) (Ashida et al., 2012). In contrast, toxins referred to as AB toxins, where “A” is the subunit with enzymatic activity and “B” is the subunit binding receptors on the cell surface, are rather internalized through endocytosis (Odumosu et al., 2010).

Despite the importance of such findings, until recently, not much attention was paid to the investigation of the capacity of bacteria to alter the host cell cycle and to the analysis of this alteration on the outcome of the infection.

## The cell cycle of eukaryotic cells and cell cycle regulation

**Figure 1:**
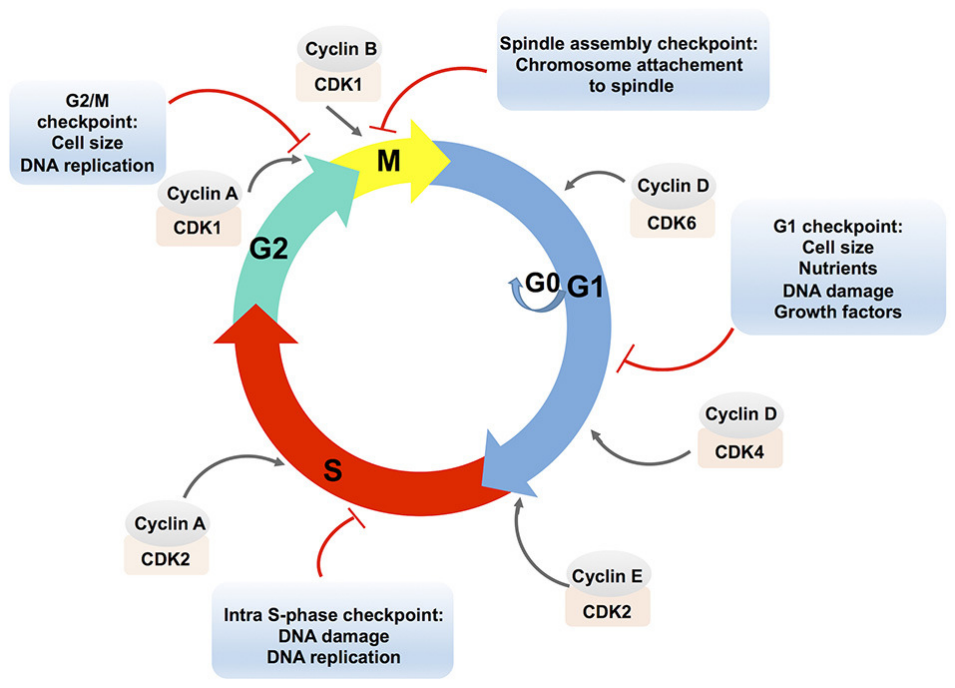
**Schematic presentation of the eukaryotic cell cycle and its regulation**. The eukaryotic cell cycle consists of two gap phases, the G1 and the G2 phase, the S-phase, and the M (mitosis) phase. Cells can also enter a quiescent state, the G0 phase. Cell cycle phases are indicated by colored arrows. The cell cycle is regulated by complexes that are composed of cyclins, which are bound to cyclin-dependent protein kinases (CDKs). Cyclin-CDK complexes are positioned in the front of the arrow that designates the corresponding cell cycle phase. Cyclin-CDK complexes are controlled via checkpoint pathways whose role is to prevent the cell from progressing to the next stage when it is not allowed. Multiple stimuli that exert the checkpoint control are indicated in an appropriate text insert.

The eukaryotic cell cycle is a ubiquitous and complex process involving DNA replication, chromosome segregation and cell division. The cell cycle consists of different phases: the gap phase 1 (G1), characterized by cell growth; the S-phase characterized by DNA replication; the gap phase 2 (G2), in which cells are prepared for division; and the mitosis (M) phase, which culminates in cell division. Cells can also exit the cell cycle and enter a quiescent state, the G0 phase (Figure 1; Vermeulen et al., 2003).

Cell cycle progression is controlled by the activities of complexes that consist of cyclins (A, B, D, E) bound to cyclin-dependent protein kinases (CDKs). The D-type cyclins activate the CDK4 and CDK6, which are required for an entry and a progression of cells into the G1-phase. To progress from the G1 to the S phase, cyclin E associates with CDK2. Cyclin A associated with CDK2 allows progression through the S phase. In the G2 phase, cyclin A associated with CDK1 triggers the entry into the M phase. Subsequently, cyclin B activates the CDK1 and promotes the M phase of the cell cycle (Lim and Kaldis, 2013).

The formation and activity of cyclin-CDK complexes are regulated by the synthesis of cyclins and their degradation during the cell cycle progression, by the CDK phosphorylation status, or by the binding of CDK inhibitory proteins to the cyclin-CDK complexes (Lim and Kaldis, 2013).

The combined effects of these pathways control the cell cycle progression in response to external stimuli as well as to the internal cell environments, e.g., through the checkpoint pathways. In addition to the modulation of the cell cycle, checkpoint pathways control DNA repair pathways, activation of transcriptional programs, and stimulation of apoptosis in case of persistent damage (Zhou and Elledge, 2000). Checkpoint arrests occur at different stages of the cell cycle: the G1/S transition (the G1 checkpoint), the S phase progression (the intra-S phase checkpoint), the G2/M boundary (the G2/M checkpoint) and the spindle checkpoint at the transition from metaphase to anaphase during mitosis (Figure 1). Checkpoint activation results either in cell death or in improved cell survival and deregulation of these critical signaling pathways may lead to the disruption of essential cellular functions. It has to be noted that the expression of many genes is cell cycle-regulated (Grant et al., 2013) and it was shown that transcriptional and post-transcriptional mechanisms control cell cycle regulators (Nath et al., 2015).

In some cases, bacterial pathogens have developed strategies to promote their colonization, with the action of virulence factors controlling the main host functions, including cell cycle. This review is designed to summarize current knowledge regarding cyclomodulins, bacterial toxins and effectors that induce eukaryotic cell cycle alterations (Nougayrede et al., 2005; Oswald et al., 2005). The role of bacterial cyclomodulins in the context of the host-microbes interaction is the emergent issue in the field of infectious diseases. While the structures and the mechanisms of the action of cyclomodulins are regularly investigated, the data regarding the analysis of the cell cycle alteration on the outcome of the infection is very scarce. We discussed bacterial virulence factors for which the consequence of their cyclomodulin activities has been described. Representative cyclomodulins with the different modes of actions are provided in the review. Particular attention is paid to the deciphering of the consequences of host cell cycle alteration.

## Bacterial cyclomodulins

Bacteria produce virulence factors including effectors injected by bacteria into the host cells and toxins released by bacteria into the surrounding medium, which hijack the main functions of host cells thus promoting bacterial invasion and host colonization. Growing evidence demonstrates that the host cell cycle progression is one of the most favorable targets of these virulence factors. Therefore, a new classification of bacterial toxins and effectors, based on their ability to alter the host cell cycle, has emerged under the term cyclomodulin (Nougayrede et al., 2005; Oswald et al., 2005). Our understanding of the capacities of cyclomodulins to alter the eukaryotic cell cycle roughly follows the investigations of their structure and the deciphering of their mechanisms of action (Ha et al., 2007; Li et al., 2007; Deplanche et al., 2015). The cyclomodulins are a heterogeneous functional class of microbial virulence factors: most cyclomodulins consist of proteins, but the existence of non-proteinaceous bacterial cyclomodulin with different mechanisms of action was also reported. Some of the cyclomodulins have enzymatic activities and they are classified as transferase, deamidase, deaminase, protease, glycosidase, phosphatase or DNAses. Among cyclomodulins, which display enzymatic activities there are AB toxins, that interact with receptors on the cell surface using one or several B-binding domains and modify the action of intracellular host targets through their enzymatic activities via A-active domain (Odumosu et al., 2010).

In addition to the cyclomodulins bearing an enzymatic activity, there are pore-forming proteinaceous cyclomodulins (e.g., PVL), peptidic cyclomodulins (e.g., PSM), as well as non-proteinaceous ones (e.g., mycolactones).

Cyclomodulins thus belong to a growing and heterogeneous family and we can expect that other bacterial molecules will enrich this family.

Up to date, there is no precise classification of bacterial cyclomodulins. We therefore proposed hereafter a presentation order based on the proteinaceous or non-proteinaceous nature of the cyclomodulins. Among proteinaceous cyclomodulins, we distinguish those bearing an enzymatic activity and the non-enzymatic ones.

### Cyclomodulins: protein toxins or peptide toxins

#### Cyclomodulins with enzymatic activities

##### Cycle inhibiting factor

Enterohemorrhagic and enteropathogenic *E. coli* strains (EHEC and EPEC, respectively) are major causes of infectious diarrhea in children worldwide. EHEC and EPEC use the Type III secretion system (T3SS) encoded by the Locus of Enterocyte Effacement (LEE) to promote their establishment within the host by delivering different virulence effectors to infected host cells (Table 1; Hsu et al., 2008). Cycle inhibiting factors (CIFs) are effectors delivered by EHEC and EPEC through T3SS. CIF is not encoded by the LEE but instead by a lambdoid prophage that has possibly been acquired through horizontal transfer (Hsu et al., 2008). CIF causes cell arrest at both the G1/S and G2/M transitions by accumulation of the CDK inhibitors, p21^Cip1^ and p27^Kip1^ (Samba-Louaka et al., 2008; Morikawa et al., 2010; Taieb et al., 2011). Such arrest is mediated by deamidation of the ubiquitin-like protein NEDD8 by CIF (Cui et al., 2010) demonstrating that CIF displays deamidase enzymatic activity. NEDD8 deamidation impairs the conjugation of NEDD8 to Cullin, which inactivates a Cullin-Ring ubiquitin Ligase (CRL) (Cui et al., 2010). The inactivation of CRLs results in inhibition of the ubiquitin-dependent degradation pathway and thus accumulation of CRLs' substrates, including p21^Cip1^ and p27^Kip1^ which induces the host cell cycle arrest (Zhou and Zhu, 2015). Cells exposed to CIF have also shown formation of stress fibers and focal adhesion (Samba-Louaka et al., 2009b) beyond the cellular and nuclear enlargement that probably result from inhibition of CRL-dependent RhoA degradation (Chen et al., 2009; Jubelin et al., 2010). In such cases, cells may restart the DNA synthesis process without proceeding to division, probably owing to the inhibition of CRL dependent degradation of the licensing factor Cdt1, thus increasing their DNA content (Nougayrede et al., 2001; Jubelin et al., 2010). Proteins homologous to CIF proteins were found in several other pathogenic Gram negative species or genera (*Yersinia pseudotuberculosis, Photorhabdus luminescens, Photorhabdus asymbiotica*, and *Burkholderia pseudomallei)* (Jubelin et al., 2009). Consistent with the fact that CIF proteins are T3 secreted effector, all these bacteria possess a T3SS. Despite the differences in primary sequences of these homologs, the catalytic site is conserved and was demonstrated to play a pivotal role in the eukaryotic cell cycle alteration and the cytoskeleton reorganization (Jubelin et al., 2009).

**Table 1:** **Cyclomodulins and their key features**.

	**Toxin type**	**Species**	**Proteins**	**Enzymatic activity**	**Cell cycle phase delay**
**PROTEIN OR PEPTIDES TOXINS**					
**Cyclomodulins with enzymatic activities**					
Cycle Inhibiting Factor (CIF)	Cysteine protease	*E. coli (EHEC, EPEC)*	2 domains: N-terminal (secretion and translocation) C-terminal (enzymatic)	Deamidase	G1/S G2/M
		*Y. pseudotuberculosis*			
		*Pseudomonas* sp.			
		*Enterobacter* sp.			
		*Serratia* sp.			
Γ-glutamyl transpeptidase (GGT)	Enzyme	*H. pylori*	1 protein with 2 chains cleaved by autocatalysis	Gamma-glutamyltransferase	G1/S
Cytolethal Distending Toxin (CDT)	Three globular subunits	*E. coli H. hepaticus S. enterica serovar Typhimurium*	CdtB catalytic subunit CdtA and CdtC cell binding subunits	CdtB subunit: Dnase and phosphatase	G1/S G2/M
Shiga toxin (Stx) (Verotoxin)	AB5 toxin	*S. dysenteriaeE. Coli (STEC)*	stxA enzymatic subunit StxB binding subunit	A subunit: N-glycosidase	S
Subtilase AB (SubAB)	AB5 toxin	*E. coli (STEC)*	SubA enzymatic subunit SubB binding subunit	A subunit: protease	G1/S
Anthrax toxin (Edema toxin/Lethal toxin)	Tripartite toxin	*B. anthracis*	Edema and/or Lethal factor (A enzymatic subunit) Protective Antigen (B binding subunit)	Edema factor : adenylate cyclase Lethal factor: zinc metalloprotease	G1/S
Cholera toxin (Ctx)	AB5 toxin Oligomeric complex	*V. cholerae*	CTA comprises CTA1 and CTA2 domains CTB (B binding subunit)	ADP-ribosyltransferase	G1/S
Adenylate Cyclase Toxin (ACT)	AB5 toxin	*B. pertussis*	S1 enzymatic A subunit S2 to S5 binding B subunits	A subunit: acetyltransferase	G1/S
Vacuolating cytotoxin (VacA)	Pore-forming toxin	*H. pylori*	3 domains (p33, p55, β-barrel)	Hypothetically	G1/S
Cytotoxic Necrotizing Factor 1 (CNF1)	Non canonical AB toxin	*E. coli*	3 domains: N-terminal (binding) C-terminal (enzymatic) Central (translocation)	Deaminase	G2/M
**Cyclomodulins without enzymatic activities**					
Panton–Valentine leukocidin (PVL)	β-pore-forming toxin Bi-component toxin	*S. aureus*	LukS-PV LukF-PV	No	G0/G1
Phenol soluble modulins (PSMs)	Peptides	*S. aureus*	PSMα, PSMβ, PSMγ	No	G2/M
**NON-PROTEINACEOUS CYCLOMODULINS**					
Mycolactone	Macrolide	*M. ulcerans*	−	No	G0/G1

##### γ-glutamyltranspeptidase

*Helicobacter pylori* is a Gram-negative bacterium that colonizes the intestinal mucosa and causes several diseases such as chronic gastritis, gastric cancer and ulcers. *H. pylori* produces a hydrolase γ-glutamyltranspeptidase, (GGT), a virulence factor that catalyzes the transpeptidation and hydrolysis of the gamma-glutamyl group of glutathione and related compounds (Table 1; Ricci et al., 2014).

*H. pylori* GGT is synthesized as a single 60 kDa precursor protein expressed by the gene *HP1118* (Shibayama et al., 2003). Following an autocleavage at Threonin 380, GGT releases two units, 40 kDa, and 20 kDa, which form the active heterodimer (Shibayama et al., 2003). The enzymatic activity of GGT resides in the 20 kDa subunit with the gamma**-**glutamyl binding site at the Tyr433, and the Arg475 residues and the C-terminus of 20 kDa subunit contributes to catalysis (Williams et al., 2009).

GGT, as a virulence factor, has gained increased attention during the past decade due to its harmful effects on the host. GGT is mainly associated with colonization and gastric ulcer induction, as was observed in some animal models (McGovern et al., 2001; Gong et al., 2010). GGT causes damage to gastric cells, including apoptosis (Shibayama et al., 2003), the production of reactive oxygen species causing DNA damage (Ding et al., 2007; Gong et al., 2010), an induction of inflammatory responses by an increase in the expression of cyclooxygenase-2 (Busiello et al., 2004) and interleukin 8 (Gong et al., 2010), as well as cell cycle delay (Kim et al., 2010). Exposure of human cancer cells AGS (gastric adenocarcinoma cells) to GGT resulted in G1 phase arrest. The arrest was associated with a decrease in the expression of cyclin E, cyclin A, Cdk 4, and Cdk 6, and with an increase in the expression of the cyclin-dependent kinase (Cdk) inhibitors p27^Kip1^ and p21^Cip1^, suggesting that GGT hampers the G1-S phase transition (Kim et al., 2010). Recently it was shown that additionally to the G1 phase arrest in epithelial cells *H. pylori* GGT induces the G1 phase arrest of T cells through the disruption of Ras-dependent signaling (Schmees et al., 2007).

##### Cytolethal distending toxin

Cytholetal distending toxin (CDT) is produced by a variety of Gram-negative bacteria such as *E. coli, Helicobacter hepaticus, Actinobacillus actinomycetemcomitans* and other bacteria (DiRienzo, 2014; Taieb et al., 2016; Table 1). Initially CDT, a member of AB_2_ toxin superfamily, was described in 1987 in a pathogenic *E. coli* strain isolated from a child (Anderson et al., 1987). CDT blocks the cell cycle of the host cell and induces DNA single and double strand breaks (DSB). It may induce an increased risk of cancer development (Anderson et al., 1987; Ge et al., 2007; Grasso and Frisan, 2015).

CDT is the product of an operon encoding three proteins: CdtA, CdtB, and CdtC. CDT is an AB_2_ type toxin in which the CdtB subunit corresponds to the A (active)-domain and exhibits a DNase activity responsible for DSB, and a phosphatase activity that resembles that of phosphatidylinositol 3,4,5-triphosphatase (Elwell and Dreyfus, 2000; Shenker et al., 2007; Guerra et al., 2011; Gargi et al., 2012; Grasso and Frisan, 2015). CdtA and CdtC, are two ricin-like lectin domains, which allow CDT to bind to the susceptible cell (B_2_-domain), leading to its internalization. CdtB is then relocated to the nucleus by a retrograde transport pathway via early and late endosomes (Guerra et al., 2005). It was shown that CdtA, CdtB, and CdtC form a ternary complex with three interdependent molecular interfaces (Nesić et al., 2004).

In contrast to other pathogens, in the *Salmonella typhi* typhoid toxin, an A_2_B_5_ toxin, CdtB is cross-linked via a disulfide bond to PltA (the pertussis-like toxin A, homologous to the pertussis toxin ADP-ribosyltransferase subunit). These two active (A) proteins are non-covalently bound with a B_5_ (binding) subunit consisting of a pentameric PltB (pertussis-like toxin B) subunit responsible for cell binding instead of being associated with CdtA and CdtC (Spanó et al., 2008; Song et al., 2013; Bezine et al., 2014).

**Figure 2:**
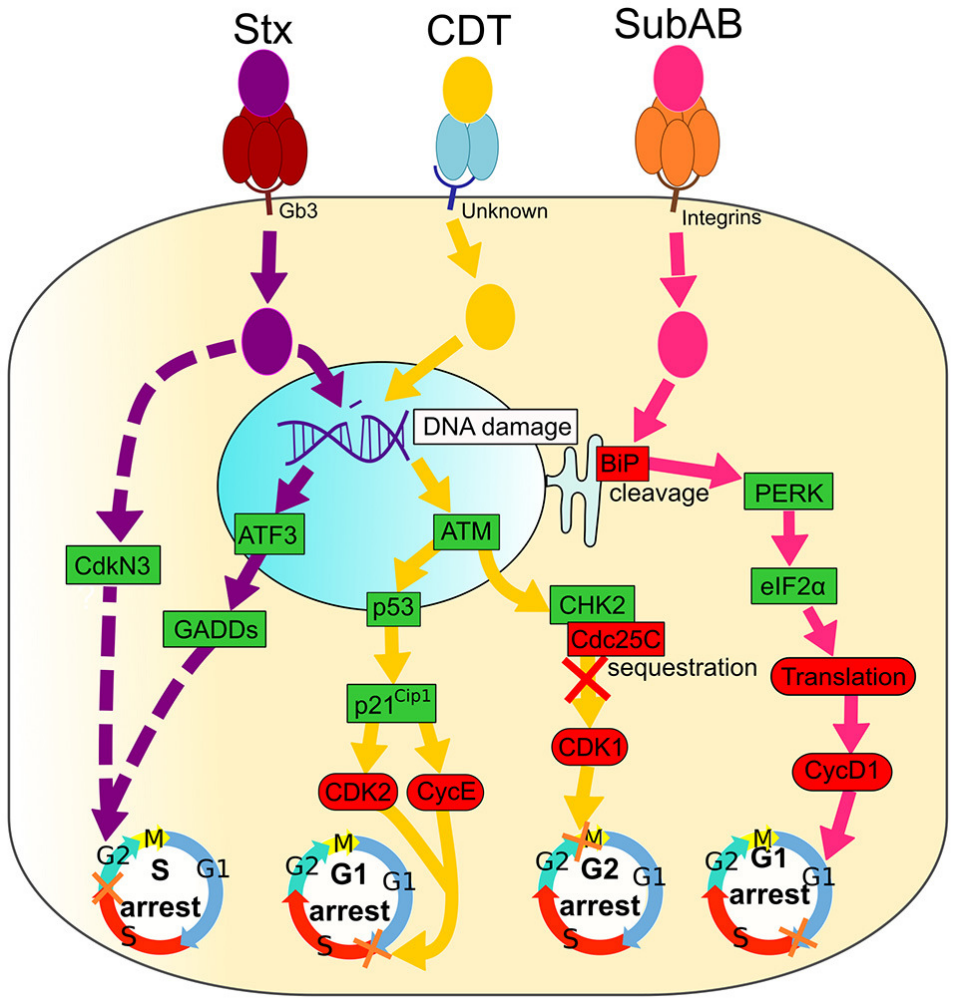
**Signaling pathways of Shiga toxin (Stx), Cytolethal Distending Toxin (CDT) and Subtilase AB (SubAB)**. Activated and inactivated proteins are colored in green and red, respectively. Arrow colors match catalytic moieties of toxins. Dashed arrows are drawn when the precise mechanism is unknown. (i) Shiga toxin (Stx) binds to the cell-surface receptor Gb3 through the pentameric B subunit (dark red), followed by an internalization of the enzymatic A subunit (purple). Stx induces irreversible DNA damage that activates ATF3 and GADDs proteins, leading to cell cycle arrest in the G2 phase. Stx also induces CdkN3 that results in cell cycle arrest in the G2 phase. (ii) Cytolethal Distending Toxin (CDT) binds to an unknown cell membrane receptor through CdtA and CdtC (blue), leading to an enzymatic CdtB (yellow) internalization. CDT causes DNA damage that leads to the activation of ATM, followed by Cdc25C sequestration by CHK2. Consequently, Cdc25C is not free to bind CDK1, leading to its inhibition and, ultimately, to arrest of cells in the G2 phase. DNA damage caused by CDT also activates p53 and p21^Cip1^, causing CDK2, and CycE inactivation and cell cycle arrest in the G1 phase. (iii) Subtilase AB binds integrins at the cell surface with the pentameric B subunit (orange) followed by the entrance of the enzymatic A subunit (pink). SubAB cleaves the chaperone BiP that activates PERK and eIF2α, leading to a translation inhibition. Finally, cyclin D1 is down-regulated and causes the arrest of cells in the G1 phase.

The CdtB subunit is highly conserved among various bacteria and has 25-40% identity with phosphodiesterase enzymes, including DNase I (Elwell and Dreyfus, 2000; Nesić et al., 2004). The nuclease activity shown by this subunit triggers a DNA damage response (DDR) (Grasso and Frisan, 2015). The activation of the DDR is initiated by the ataxia telangiectasia mutated (ATM) kinase that phosphorylates CHK2, a serine/threonine kinase that, in turn, phosphorylates various cell cycle regulators such as phosphatases Cdc25A and Cdc25C (Figure 2; Bezine et al., 2014). Phosphorylation of Cdc25C creates an interaction site for the 14-3-3 family of proteins that sequester Cdc25C in the cytoplasm (Ahn et al., 2004). Thus, phosphatase Cdc25C is unable to activate the nuclear hyper-phosphorylated (inactive) CDK1/cyclin B complex, leading to the delay in the G2/M phase transition of the cell cycle (Taieb et al., 2015). Delay at the G1/S phase by CDT is also reported, and results from an ATM/p53-dependent accumulation of the CDK2-cyclin E inhibitor, p21^Cip1^. Inhibition of CDK2-cyclin E blocks the entry into the S phase of the cell cycle (Cortes-Bratti et al., 2001; Ge et al., 2008).

Evidence indicates that CDT is a beneficial virulence factor for bacterial survival (Scuron et al., 2016). It was found that CDT increases bacterial gut colonization, promotes pro-inflammatory responses, and deregulates immune response through an alteration of cytokine synthesis that may in turn increase the local inflammation (Akifusa et al., 2001; Ge et al., 2008; Jain et al., 2008; Belibasakis and Bostanci, 2014). Moreover, it was shown that CDT of *A. actinomycetemcomitans* interferes with bone metabolism notably by affecting the signaling of osteoclast differentiation at the site of the infection (Belibasakis et al., 2005a,b, 2008).

The effect caused by CDT may lead to apoptosis or necrosis of cells through the mitochondrial effectors Bax/Bcl-2, cytochrome C release and caspase activation (Liyanage et al., 2010). The consequence of the genotoxic effects of CDT is related to the induction of cellular senescence associated with persistently activated DNA damage signaling that may result in genomic instability (Bezine et al., 2014; Graillot et al., 2016).

Colibactin is another well-known cyclomodulin produced by *E. coli* and as the CDT genotoxin, induces host DNA strand breaks and activates the DNA damage response. The mode of action of colibactin has been previously described in numerous publications (Balskus, 2015; Rosadi et al., 2016; Taieb et al., 2016).

##### Shiga toxin

Shiga toxins (Stxs) are produced by *Shigella dysenteriae* serotype 1 bacteria and *E. coli*, capable of producing Shiga toxin type 1 (Stx1), type 2 (Stx2), or both, encoded by *stx1* and *stx2* genes, respectively (Table 1; Paton and Paton, 1998; Johannes and Romer, 2010). Cytotoxin from culture filtrates of some diarrhea-inducing *E. coli* strains similar to Shiga toxin was able to kill Vero cells, consequently this toxin was also named verotoxin. These Shiga toxin-producing *E. coli* (STEC) or Verotoxin-producing *E. coli* (VTEC) produce the same toxin, known as Shiga toxin or Verotoxin (Lee et al., 2016). S. *dysenteriae* and STEC are major public health concerns in developed and developing countries since they can induce hemorrhagic colitis or hemolytic-uremic syndrome (Farrokh et al., 2013). Stx belongs to the family of AB_5_ toxins that consist of the ribosomal 32 kDa RNA-cleaving A subunit, which is non-covalently bound to five receptor-binding 7.7 kDa B subunits (Figure 2; Melton-Celsa, 2014). Once bound to the host cells receptor (Gb3), the toxins are internalized and undergo retrograde intracellular trafficking: they are transported from an early endosome, through the Golgi apparatus, to the endoplasmic reticulum (ER) (Sandvig et al., 2010). During this retrotranslocation, the A subunit dissociates from the B subunit due to a proteolysis and a disulfide bond reduction (Melton-Celsa, 2014). Once in the cytoplasm, the A subunit acquires a native conformation and activates its enzymatic features, leading to the removal of an adenine residue from the ribosomal RNA of eukaryotic cells and, subsequently, to an impaired protein synthesis (Melton-Celsa, 2014).

In addition to the protein synthesis inhibition, Stx induces the ribotoxic stress response and ER stress that may result in apoptosis, autophagy or an activation of the innate immunity associated with a cytokine/chemokine production that contribute to tissue damage in multiple organs (Jandhyala et al., 2008; Lee et al., 2016). It was shown that *Stx* induces an arrest of epithelial cells in the S phase of a cell cycle (Figure 2), which is a consequence of irreversible DNA damage (Bhattacharjee et al., 2005). The delay in the S phase is associated with an up-regulation of genotoxic stress response genes both at mRNA and protein levels. The level of the ATF3 (Activating Transcription Factor), three GADD (Growth Arrest and DNA Damage) family genes (GADD45a, GADD45b, and GADD34), and the cell cycle-related phosphatase CdkN3 (Bhattacharjee et al., 2005), which is an inhibitor of CDKs (Nalepa et al., 2013) is increased in Stxs-treated cells.

##### Subtilase toxin

Subtilase toxin (SubAB) is an AB_5_ type toxin that is produced by STEC (Table 1; Paton and Paton, 2010). The *in vivo* effects of SubAB have been examined in mice and were shown to cause microvascular injury, thrombosis and necrosis in various organs such as the brain, the liver and the kidneys (Paton et al., 2004). SubAB may contribute to the pathogenesis of a systemic hemolytic uremic syndrome.

Similar to other AB toxins, five 13 kDa B subunits of SubAB bind to the host cells receptors, while the 35 kDa A subunit possesses an enzymatic activity that is essential for cytotoxicity (Michelacci et al., 2013). Once internalized into the cells by endocytosis, SubAB trafficks through Golgi to the ER (Morinaga et al., 2008). SubAB induces ER stress and cleaves the chaperone BiP/Grp78 in its carboxy-terminal portion, localized in lumen of ER. BiP/Grp78 is essential for the proper folding and the assembly of nascent proteins (Figure 2; Nours et al., 2013). This results in the inhibition of protein synthesis (Morinaga et al., 2008). SubAB-induced ER stress caused many other cellular events such as transient phosphorylation of Akt and activation of NF-κB signaling, a down-regulation of gap junction expression and an induction of apoptosis (Yahiro et al., 2014).

The cleavage of BiP/Grp78 was associated with a phosphorylation of a double-stranded RNA-activated protein kinase-like ER kinase (PERK) and a eukaryotic initiation factor-2α (eIF2α) (Morinaga et al., 2008). A degradation of BiP/Grp78 leads to cyclin D1 down-regulation caused by both a SubAB-induced translational inhibition and a continuous proteasomal degradation, and results in G1 phase arrest (Figure 2; Morinaga et al., 2008; Yahiro et al., 2012; Márquez et al., 2014).

##### Anthrax toxin

*Bacillus anthracis* is the causative agent of anthrax, a disease with local or systemic clinical manifestations. Cutaneous anthrax is characterized by edematous necrotic lesions that become black eschars, while systemic anthrax has a multiplicity of symptoms, including of hypotension and shock, followed by sudden death (Ascenzi et al., 2002).

The *B. anthracis* toxins are in fact a tripartite toxin, which belongs to the AB toxin family. It consists of the heptameric protective antigen (PA) (B subunits) and two alternative A subunits: the lethal factor (LF), forming lethal toxin (LT), and the edema factor (EF), forming edema toxin (ET) (Table 1; Friebe et al., 2016).

**Figure 3:**
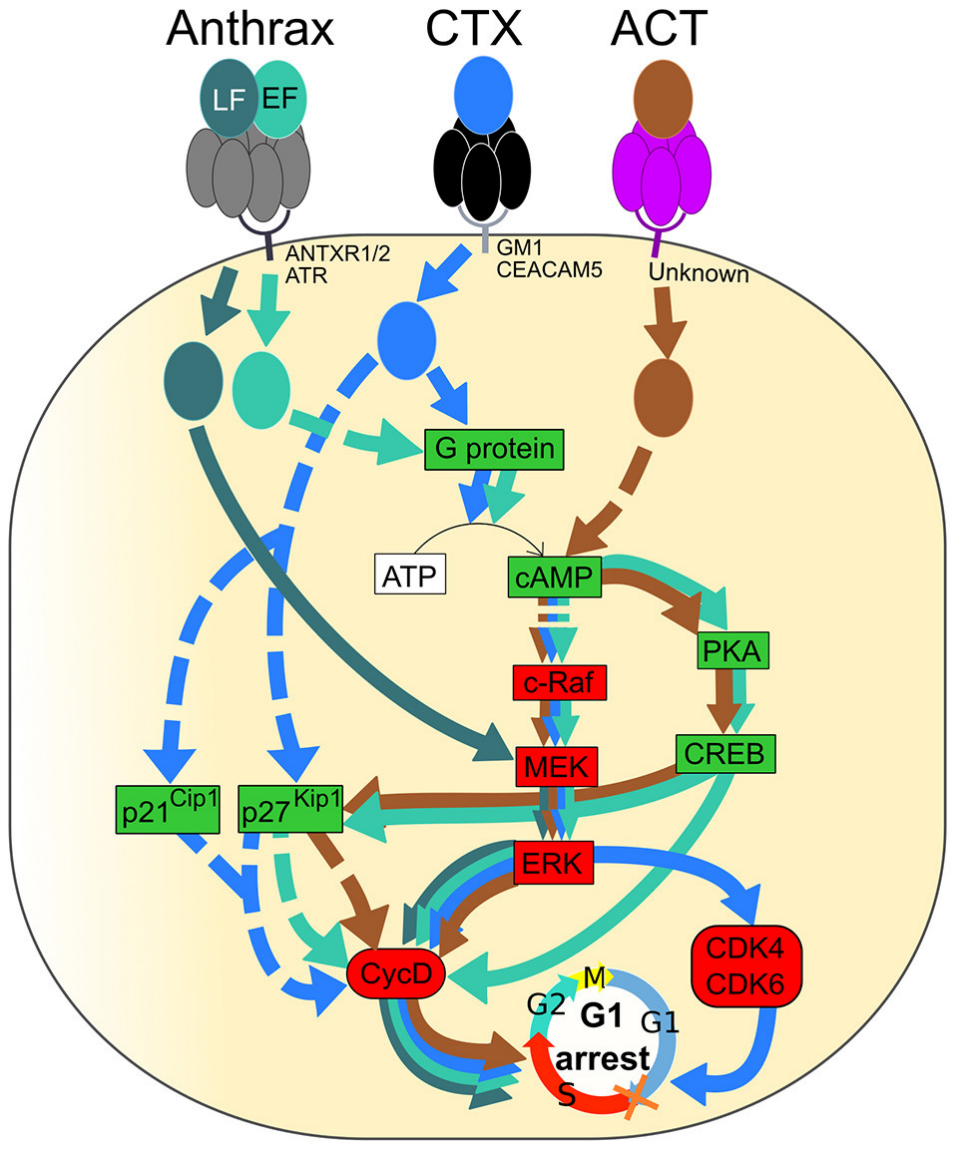
**Signaling pathways of Anthrax toxin, Cholera toxin (CT) and Adenylate cyclase toxin (ACT) resulting in G1 phase arrest of the eukaryotic cell cycle**. Activated and inactivated proteins are colored in green and red, respectively. Arrow colors match catalytic moieties of toxins. Dashed arrows are drawn when the precise mechanism is unknown. (i) Anthrax toxin is formed by EF, LF, and PA. Heptameric PA (gray) binds to either ANTXR1, ANTXR2, or ATR at the cell membrane and leads to the entrance of EF and LF into the cell. EF (green) induces cAMP production followed by inactivation of the c-Raf/MEK/ERK cascade, leading to Cyclin D1 inactivation. A cAMP increase induces PKA and CREB, leading to cyclin D1 inactivation. The level of p27^Kip1^ is increased by cAMP and leads to cyclin D and, especially, Cyclin D1 inactivation. LF (dark green) directly inactivates MEK, leading to ERK and Cyclin D1 inactivation. (ii) Cholera toxin (CTX) binds to either GM1 or CEACAM5 at the cell membrane through the pentameric CTB subunit (black), leading to endocytosis of the catalytic CTA subunit (blue). CTX activates the G protein and leads to cAMP production followed by up-regulation of p27^Kip1^ and p21^Cip1^ and inactivation of the c-Raf/MEK/ERK cascade, leading to Cyclin D1, CDK4, and CDK6 inactivation. (iii) Adenylate cyclase toxin (ACT) binds to an unknown receptor at the cell surface through the pentameric subunit (purple), and the catalytic subunit (brown) is translocated to the cytosol. In the same way as EF, ACT induces production of cAMP that leads to inactivation of the c-Raf/MEK/ERK cascade, activation of PKA and CREB, activation of p27^Kip1^ and, finally, inactivation of Cyclin D1. Inactivation of Cyclin D1, CDK4, and CDK6 leads to cell cycle arrest in the G1 phase.

Using *in vivo* mice model it was shown that subcutaneous administration of ET results in skin edema, while intravenously injected ET can directly target intestinal epithelial cells and hepatocytes and has been shown to be lethal through its action in hepatocytes (Moayeri et al., 2015). The 83 kDa PA binds to protein receptors on the host cells, such as tumor endothelial marker 8 (TEM8), also known as anthrax toxin receptor 1 and capillary morphogenesis gene product 2 (CMG2), also known as anthrax toxin receptor 2, followed by the entrance of the catalytic moieties of the toxins, EF or LF, into the host cell cytoplasm (Bradley et al., 2001; Scobie et al., 2003). EF is an adenylate cyclase that, upon translocation into host cells and after association with the calcium-binding protein, calmodulin, catalyzes the production of a ubiquitous second messenger cyclic AMP (cAMP), resulting in the impairment of the cell homeostasis through multiple changes (Figure 3; Leppla, 1982; Tang and Guo, 2009).

The proliferation of many cells is controlled by the phosphorylation of extracellular signal-regulated kinase ERK due to the action of mitogen-activated protein kinase (MEK) that communicates a signal from cellular receptors to the nuclear DNA, thereby regulating the gene expression; this transduction pathway is influenced by cAMP (New and Wong, 2007). EF-induced cAMP production reduces the amount of the phosphorylated ERK and stimulates phosphorylation of the Cyclic AMP Response-Element Binding (CREB) protein in part through cAMP-dependent Protein Kinase A (PKA) (Gray and Hewlett, 2011). EF-induced cAMP accumulation leads to a decrease in the level of cyclin D1 that, together with CDKs, regulates the entry into the G1 phase. The level of p27^Kip1^, which inhibits the complex of cyclin D and CDKs, is augmented due to the cAMP increase. Consequently, an accumulation of cells in the G1/G0 phase and a decrease of cells in the S phase occurs in ET-treated cells (Gray and Hewlett, 2011).

LF, the other catalytic subunit of the anthrax toxin, is a zinc metalloprotease that cleaves and inactivates the N-terminal end of MEKs. It leads to the inhibition of ERK, followed by a down-regulation of cyclin D1, cyclin D2 and checkpoint kinase 1 (Ha et al., 2007). The important role of cyclin D1 and D2 for escaping from G1 and initiation and completion of S phase through activating cyclin-dependent kinases 2, 4, and 6 has been documented (Sanchez and Dynlacht, 2005). Those events result in the arrest of cells in the G1/G0 phase of the cell cycle (Figure 3; Ha et al., 2007). Ultimately an LF-induced cleavage of MEK, which would lead to impairment of cyclin function and homeostasis, promotes a belated induction of cell death via a transcriptome deregulation of host factors, resulting in its cytotoxicity on human endothelial cells (Rolando et al., 2010).

##### Cholera toxin

The cholera toxin (CT or CTX) is the major virulence factor of *Vibrio cholera* and the main diarrhea-causing enterotoxin (Table 1). CTX is a member of the AB_5_ toxin family that consist of a cell surface receptor-binding homopentameric B subunit (CTB) that is linked to a catalytic A subunit (CTA). CTA comprises the CTA1 domain, which activates G proteins and CTA2 domain (Figure 3; Sánchez and Holmgren, 2011). The pentameric CTB subunit binds to GM1 gangliosides receptors of target cells or to other types of glycans. A binding of the pentameric CTB subunit to the GM1 ganglioside receptor on the intestinal cells (enterocytes) triggers CTX endocytosis followed by the cleavage of CTA1, which then becomes an active enzyme. Once activated, CTA1 catalyses ADP-ribosylation of the Gs alpha subunit (Gα_*s*_) of the G protein, thereby stimulating adenylate cyclase to produce cAMP (Figure 3). The high cAMP levels impede the electrolyte balance, causing a drastic efflux of ions and water from enterocytes, leading to watery diarrhea (Nichols et al., 2015).

CTX induces the G1-phase arrest in eukaryotic cells. The capacity of CTX to modulate the cell cycle progression of C6 rat glioma cells was reported by Li et al. (2007). CTX induces an accumulation of cells in the G1 phase due to the down-regulation of cyclin D1 and CDK2 proteins along with an up-regulation of the cell-cycle inhibitory proteins, p21^Cip1^ and p27^Kip1^ (Figure 3; Li et al., 2007). Moreover, using two human bladder cell lines, T24 and UM-UC-3, it was demonstrated that CTX inactivates the c-Raf/Mek/Erk cascade, via the PKA-dependent c-Raf phosphorylation at Ser-43. This results in the downregulation of the expression of the regulatory molecules (cyclin D1, Cdk4, and Cdk6), followed by the G1 phase arrest (Figure 3; Zheng et al., 2014).

##### Adenylate cyclase toxin

*Bordetella pertussis*, a Gram-negative bacterial pathogen, is responsible for respiratory infections manifested by whooping cough, with possible lethal complications (Table 1). Similar to *B. anthracis, B. pertussis* produces an adenylate cyclase toxin (ACT), which belongs to the AB_5_ toxin family (Figure 3; Melvin et al., 2014). ACT of *B. pertussis* is a ~200 kDa protein consisting of two functional domains: an N- terminal adenylate cyclase enzyme domain (AC domain) and a pore-forming or hemolysin domain (Hly domain), which belongs to the RTX (Repeats in Toxin) family (Carbonetti, 2010). ACT displays the hemolytic/pore-forming activity along with the adenylate cyclase enzymatic activity (Basler et al., 2006). ACT is released by the Type I bacterial secretion system (Glaser et al., 1988). The Hly domain is required for the delivery of the AC domain into the cell cytosol either via binding to the α_m_β_2_ integrin (CD11b/CD18) as a cell receptor or by direct translocation to the eukaryotic cells cytosol (Guermonprez et al., 2001; Eby et al., 2010).

Once reaching the cytosol, the AC domain is activated through binding of calmodulin (the mammalian cytosolic protein) and it catalyses the conversion of intracellular ATP into cAMP, the key second messenger signaling molecule (Basler et al., 2006; Kamanova et al., 2008).

It was reported that the ACT-induced cAMP signaling and ATP depletion together with pore-forming activity, can synergize in promoting of apoptosis or necrosis of phagocytes (Khelef and Guiso, 1995; Bachelet et al., 2002). An analysis of the effects of *B. pertussis* on the regulatory pathways controlling the cell cycle reveals that ACT stimulates PKA, which activates CREB through its phosphorylation that, in turn, decreases the Cyclin D level, followed by G1 phase arrest (Gray and Hewlett, 2011). On the other hand, *B. pertussis* ACT-induced cAMP via the c-Raf-MEK complex may inhibit ERK phosphorylation, resulting in decrease of the Cyclin D level and, consequently, causing a block in cell cycle progression at the G1-S transition (Gray and Hewlett, 2011; Figure 3). Furthermore, ACT may increase the cyclin-dependent kinase inhibitor, p27Kip1. Such a regulatory profile causes an accumulation of infected cells in the G1/G0 phase and a reduction of cells in the S phase (Gray and Hewlett, 2011; Figure 3).

##### Vacuolating cytotoxin

One of the major virulence factors reported for *H. pylori* is vacuolating cytotoxin A (VacA), which is known for its ability to induce the formation of large acid vesicles in the cytoplasm of gastric cells (Table 1; Cover and Blaser, 1992; Palframan et al., 2012). Cytoplasmic vacuolation is caused by the osmotic swelling of late endocytic compartments related to a VacA-dependent increase in anionic permeability. The formation of anion-conducting channels in intracellular membrane can help *H. pylori* colonization by allowing the efflux of metabolic substrates, which provides a selective advantage to toxin-producing strains in the nutrient-poor environment of the gastric mucous layer (Mendz et al., 1994).

VacA is synthesized as a 140 kDa pre-protoxin, which undergoes proteolytic processing during secretion, resulting in a mature 88 kDa monomer (Junaid et al., 2016). Intracellular-acting VacA exhibits the characteristics of an AB toxin in which, however, the enzymatic activity of subunit A is substituted by the pore-forming activity (Boquet and Ricci, 2012). The efficiency of the pore-forming activity inside the host cell is as potent as the enzymatic activity of canonical AB toxins. VacA actually targets the inner mitochondrial membrane, whose alteration is lethal for the cell (Wang and Youle, 2009). It has been shown that once inside gastric epithelial cells, VacA inserts itself into mitochondria, forms membrane channels and modulates the mitochondrial membrane permeability (Kimura et al., 1999; Kim and Blanke, 2012). This results in cytochrome C release and the execution of apoptosis through an increase in the expression of cell cycle/apoptosis regulators such as p53, p21^Cip1^ and Bax, beyond caspases-8 and -9, as was shown *in vitro* in gastric epithelial AGS cells (Manente et al., 2008; Palframan et al., 2012).

An exposure of cells to a recombinant VacA was associated with the inhibition of cell growth and resulted in morphological changes and DNA fragmentation; a cell cycle analysis revealed a prolongation of the cell cycle progression in the G1 phase (Kimura et al., 1999; Cho et al., 2003). These findings indicate that VacA of *H. pylori* induces apoptosis in gastric epithelial cells and suggests that VacA might mediate the development of gastric diseases through a cell cycle arrest in the G1 phase (Cho et al., 2003). Additionally, it was found that VacA can efficiently block the proliferation of T cells by inducing an arrest during the G1/S phase transition (Gebert et al., 2003). This influences the T cell receptor/interleukin-2 (IL-2) signaling pathway through the Ca^2+^-calmodulin-dependent phosphatase calcineurin, resulting in down-regulation of IL-2 transcription.

##### Cytotoxic necrotizing factor

The Cytotoxic Necrotizing Factor 1 (CNF-1) is a toxin produced by certain pathogenic *E. coli*. This toxin is associated with urinary tract infections as well as with skin and soft tissue infections, furthermore, CNF-1 produced by some pathogenic strains causes diarrheal illness (Table 1; Knust and Schmidt, 2010). *E. coli* CNF-1 is a 113.8 kDa AB toxin comprising an N-terminal receptor-binding domain, a binder domain with two hydrophobic helices involved in membrane translocation and a C-terminal catalytic domain (Kouokam et al., 2006; Lemonnier et al., 2007).

The first description of CNF-1 was associated with its ability to cause multinucleation, *in vitro*, in different cell lines (Caprioli et al., 1984). Investigations of molecular mechanisms of the action of CNF-1 demonstrated that CNF-1 enters the cells by receptor-mediated endocytosis, which is independent of clathrin and of sphingolipid-cholesterol-rich membrane microdomains (Contamin et al., 2000). It was found that CNF-1 activates proteins belonging to the family of Rho GTPases (e.g., Rho, Rac, and Cdc42), by promoting deamidation of glutamine 63 of RhoA or the equivalent Q61 of Rac1 and Cdc42 and converts this residue into a glutamic acid (Flatau et al., 1997; Schmidt et al., 1997; Lerm et al., 1999). To target Rho GTPases, which are located in the cytosol and at the plasma membrane, the catalytic domain of CNF-1 has to be released from the endosomal membrane. Unlike other AB-toxins, autocatalytic cleavage was not identified, indicating that endosomal protease may be involved in CNF-1 processing (Knust et al., 2009; Knust and Schmidt, 2010). Beside a pivotal role in the regulation of intercellular junctions, in the coordination of the cell motility and in the adherence of polymorphonuclear leukocytes to epithelia, Rho GTPases, when activated, are essential for the internalization of apoptotic cells and for the induction of micropinocytosis (Fiorentini et al., 1995, 2001; Hofman et al., 2000; Wang et al., 2003; Knust and Schmidt, 2010).

Moreover, it was shown that CNF-1 prevents the CDK1-Cyclin B1-dependent progression of cells from the G2 phase to the M phase in uroepithelial cells (Falzano et al., 2006). It is recognized that Cyclin B1 expression varies during the cell cycle progression with the highest level in the G2/M phase. Cyclin B1 is essentially found in the cytoplasm region through the G2 phase until it translocates into the nucleus, which precedes the nuclear envelope breakdown (Falzano et al., 2006). It was demonstrated that CNF-1 reduces cyclin B1 expression and induces a sequestration of cyclin B1 in the cytoplasm that results in CDK1 inhibition and G2/M phase arrest (Falzano et al., 2006; Giamboi-Miraglia et al., 2007).

As in case of other cyclomodulins, CNF-1-induced G2/M arrest, which impairs epithelial layer turnover, might favor *E. coli* colonization and represent a long-term risk of carcinogenesis due, to some extent, to the capacity of Rho GTPases to promote a great number of events such as motility of tumorigenic cells, metastasis, cell invasiveness and abrogation of cytokinesis (Malorni and Fiorentini, 2006).

#### Cyclomodulins without enzymatic activity

##### Panton-valentine leukocidin

*Staphylococcus aureus* (*S. aureus*) is an opportunistic pathogen responsible for a wide panel of diseases ranging from skin and soft tissue infections to life-threatening systemic diseases in humans and animals. *S. aureus* frequently promotes infections by producing powerful toxins. Panton–Valentine leukocidin (PVL) is one of the β-pore-forming toxins associated with the increased virulence of certain *S. aureus* strains (Table 1). PVL is responsible for leukocyte destruction and necrotic hemorrhagic pneumonia, a highly lethal infection that essentially affects healthy children and young adults.

PVL is a bi-component toxin that acts due to the synergistic activity of two protein subunits, 33 and 34 kDa in size, denoted as S and F according to their slow or fast elution in cation-exchange carboxy-methyl cellulose chromatography (Bronner et al., 2004). These proteins are encoded by two co-transcribed genes, *lukS-PV* and *lukF-PV*, which are located in a prophage (McClure et al., 2006). LukS-PV and LukF-PV subunits with a beta-barrel structure acquire pore-forming conformation after binding to the C5A receptor (Spaan et al., 2013), followed by hetero-oligomerization in the plasma membrane of the host defense cells. An insertion of pores into the plasma membrane leads to ion influx and efflux followed by cell lysis (Aman and Adhikari, 2014).

It was observed that the LukS-PV subunit alone inhibits the proliferation of the human acute myeloid leukemia cell line THP-1 (Bu et al., 2013). The analysis of the cell cycle of LukS-PV-treated THP-1 cells showed cell cycle changes: LukS-PV reduced the number of S-phase cells while increasing the number of G0/G1-phase cells. Moreover, LukS-PV significantly inhibited the expression of cyclin D1 that regulates the cell cycle progression from phase G1 to phase S (Lim and Kaldis, 2013). Collectively, these data suggest a LukS-PV-induced G0/G1 arrest.

##### Phenol soluble modulins

The phenol-soluble modulin (PSM) peptides were first identified in 1999 as a “pro-inflammatory complex” isolated by hot phenol extraction from *S. epidermidis* (Mehlin et al., 1999). PSMs have recently emerged as a novel toxin family that contributes to increased virulence and the spread of highly aggressive *S. aureus* isolates (Table 1; Otto, 2012; Peschel and Otto, 2013; Deplanche et al., 2016). Formed by amphipathic peptides arranged in alpha helices, PSMs are classified according to their size: short (20–25 amino acids) α-type peptides (PSMα1–PSMα4) and δ-toxin, and long (44 amino acids) β-type peptides (PSM β1 and PSM β2) (Otto, 2014).

PSMs are encoded at three different locations in the genome. Four PSMα1–PSMα4 peptides are encoded in the *psm*α operon; PSMβ1 and PSMβ2 are encoded in the *psm*β operon; and δ-toxin is encoded within the coding sequence for RNAIII, the effector of the accessory gene regulator (Agr) quorum-sensing system (Novick, 2003; Peschel and Otto, 2013).

PSMs have a multiplicity of biological functions that are essential to staphylococcal pathogenesis (Otto, 2012). PSMs trigger inflammatory responses such as chemotaxis and a priming of human neutrophils, and induce cytokine expression (Wang et al., 2007). It was found that PSMs inhibit interleukin expression during long-term infections, suggesting the involvement of PSMs in infection persistence (Deplanche et al., 2016). PSMs lyse white and red blood cells (Cassat et al., 2013) and contribute to the formation of biofilms (Periasamy et al., 2012). PSMs also present antimicrobial and immunomodulatory properties (Wang et al., 2007; Kretschmer et al., 2010; Joo et al., 2011).

We have demonstrated that the methicillin-resistant *S. aureus* MW2 induced both a decrease in the mitotic index and a cytopathic effect in host cells (Alekseeva et al., 2013). Moreover, we have shown that MW2 induces a G2/M phase transition delay in host cells, which was associated with the accumulation of the cyclin-dependent kinase Cdk1/cdc2 and unphosphorylated histone H3. Recently, we determined that PSMs were responsible for the G2/M phase transition delay, suggesting that PSMs belong to a family of cyclomodulins. The PSMα-induced G2/M phase delay was associated with an increased infective efficiency including bacterial internalization and bacterial intracellular multiplication, as well as with an decreased production of antibacterial peptides by the host cells (Deplanche et al., 2015).

### Non-proteinaceous cyclomodulins

#### Mycolactone

The opportunistic pathogen *Mycobacterium ulcerans* is responsible for Buruli ulcer (BU), an infectious disease, which is characterized by tissue necrosis and immunosuppression (Demangel et al., 2009). The macrolide mycolactone produced by *M. ulcerans*, was found to be the necessary and sufficient virulence factor for BU pathology (Table 1). Mycolactone consists of two polyketide chains, one of which forms the core lactone, a 12-member lactone ring, resulting in spontaneous cyclization (Fidanze et al., 2001).

Mycolactone-induced immunosuppression was found to be associated with the prevention of the translocation of proteins that pass through the endoplasmic reticulum before secretion (Sarfo et al., 2016). Consequently, this effect impairs the immune functions of various host cells such as monocytes, macrophages, T cells and dendritic cells that interact with mycolactone during an infection (Phillips et al., 2009; Simmonds et al., 2009). It was reported that the core lactone was sufficient for cytotoxicity of mycolactone, while the fatty acid side chain allows mycolactone to enter the host cells, enabling its interaction with intracellular target molecules (Mve-Obiang et al., 2003).

It was found that Wiskott-Aldrich syndrome protein (WASP) and neural WASP (N-WASP) are molecular targets of mycolactone (Guenin-Macé et al., 2013). WASP and N-WASP are members of a family of scaffold proteins, transducing a variety of signals and triggering the remodeling of the actin cytoskeleton (Thrasher and Burns, 2010). Mycolactone activates WASP and N-WASP through the disruption of the intermolecular interaction by a variety of ligands including the cell cycle regulator CDC42. Activation of WASP and N-WASP with a low dose mycolactone was associated to a cell cycle arrest in the G0/G1 phase resulting in apoptosis (George et al., 2000; Gama et al., 2014). The cell cycle and apoptosis are indeed interrelated: cells, which detect an alteration of cell cycle progression, can undergo apoptosis.

## Alteration of the host cell cycle as a beneficial feature for bacterial fitness

**Figure 4:**
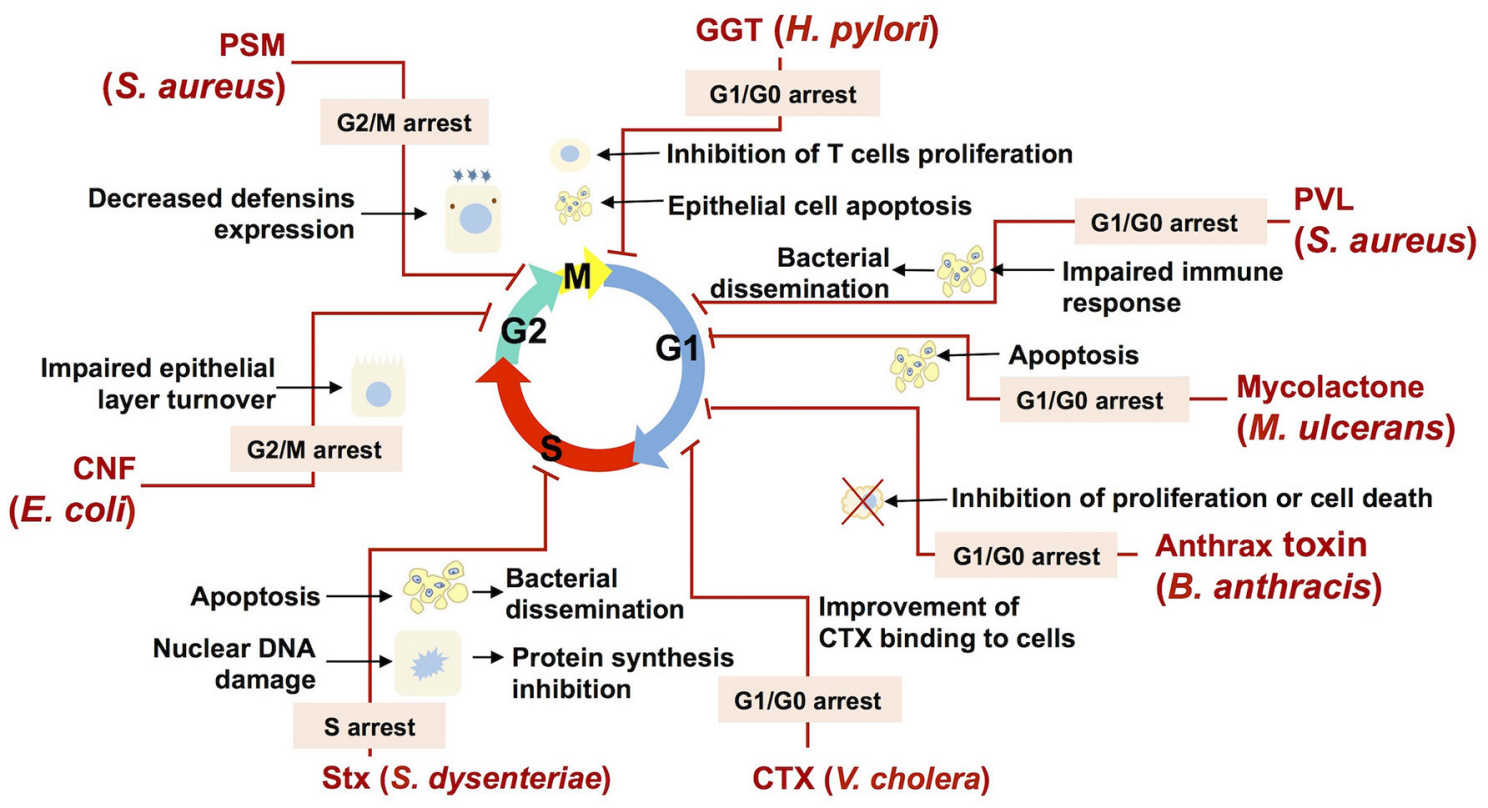
**Bacterial cyclomodulins alter the eukaryotic cell cycle for their own benefit**. The names of bacteria and the abbreviations of cyclomodulins produced by those bacteria are indicated in red. Cell cycle phases are indicated by colored arrows. Zigzag red lines are attached to the phase in which the cell cycle is blocked by the corresponding cyclomodulin. The phases in which the cell cycle is blocked are indicated in an appropriate text insert. Black arrows display the biological effects related to the cyclomodulin-induced cell cycle arrest.

Cyclomodulins present a wide array of action on diverse cellular transduction pathways, and thus would provide cyclomodulin-producing bacteria with a noteworthy fitness advantage against a eukaryotic host defenses as well as against bacterial competitors for the colonization site. Figure 4 presents a few examples of such cyclomodulin actions that are discussed hereafter.

Thus, *E. coli*-produced toxin, CNF, which is shown to induce the G2/M transition delay during infection, probably impairs the host epithelial layer turnover and, therefore, favors bacterial colonization (Figure 4; Falzano et al., 2006). Another *E. coli* toxin, CIF, which is produced by EPEC, EHEC and other (entero) pathogenic strains, induces the arrest of the G2 and the G1 phases of the cell cycle, which is correlated with the accumulation of the CDK inhibitors, p21^cip1^ and p27^kip1^. These CDK inhibitors are involved in cell cycle exit, differentiation, carcinogenesis and survival. Consequently, it is hypothesized that CIF prolongs both bacterial attachment and local persistence by slowing down a crypt–villus cell renewal (Taieb et al., 2006; Samba-Louaka et al., 2009a). Recently, it was shown that CIF is a *bona fide* virulence factor as its mutation attenuates the pathogenic potential of *Yersinia pseudotuberculosis* in a mouse infection model by blocking the bactericidal activity of Perforin-2 (McCormack et al., 2015). *H. pylori* GGT, which induces the G1 phase arrest, can trigger apoptosis in gastric epithelial cells, which in turn may result in impaired protective functions of gastric epithelium and contribute to gastric injury during *H. pylori* infection (Ricci et al., 2014). In addition to epithelial cells, it was shown that T cells are also subject G1 phase arrest, suggesting that GGT may contribute to immune evasion during *H. pylori* infection through the modulation of T cell-mediated immunity (Figure 4; Schmees et al., 2007).

The *M. ulcerans* mycolactone induces a G0/G1 cell cycle phase arrest via a remodeling of the actin cytoskeleton resulting in apoptosis (George et al., 2000; Gama et al., 2014). Mycolactone-induced apoptosis likely play a role in BU pathology. In particular, the release of internalized bacteria from apoptotic cells will prone to a bacterial dissemination within the host (George et al., 2000). Moreover, mycolactone–induced apoptosis of human peripheral blood leukocytes leads to the depletion of T cells resulting in a suppression of a local immune response (Figure 4; Fraga et al., 2011).

*S. aureus*, which commonly infects epithelial surfaces and leads to abscess formation, necrosis and a compromised integrity of the host epithelial barrier, is also known to cause morphological changes in epithelial cells such as cell enlargement and increase in nucleus size, and decrease of host cell proliferation (Alekseeva et al., 2013). An *S. aureus*-induced decrease of the host cell proliferation was associated with the G2/M transition delay, which was triggered by PSMs (Deplanche et al., 2015). A PSMα-induced delay results in an augmented staphylococcal internalization and in an enhanced intracellular proliferation, which have been shown to be more effective in G2/M phase cells than in asynchronous cells. Moreover, a PSMα-induced delay correlates with an alteration in defensins (antibacterial peptides) gene expression, with a lower expression when the cells were in the G2/M phase. This reduction of the antibacterial functions of epithelial cells demonstrates the advantage of a PSMα-induced cell cycle delay for the infection of host cells by *S. aureus* (Deplanche et al., 2015). Beyond this particular example, cell cycle arrest is likely to be used by many bacteria in order to inhibit the host defense mechanisms (Figure 4).

Leukocidin, an another *S. aureus* toxin, was shown to induce a cell cycle arrest followed by apoptosis (Bu et al., 2013) of a THP-1 cells, the common model to study monocyte and macrophage activities (Chanput et al., 2014). This finding suggests that leukocidin may be involved in the modulation of the immune response during *S. aureus* infection via a destruction of monocytes and macrophages, which favors bacterial dissemination (Figure 4). The capacity of anthrax toxin, produced by *B*. *anthracis*, to inhibit cell cycle progress in melanocytes (Koo et al., 2002) and monocytic cell lines (Kassam et al., 2005) may also impair defense mechanisms and contribute to the survival and the proliferation of *B. anthracis* within the host (Figure 4).

Bacteria can inhibit host defense mechanisms via a modification of cytokines production induced by the alteration of cells proliferation and differentiation. It was indeed shown that *H. pylori* VacA toxin induces G1/S arrest in T lymphocytes and that it results in a down regulation of cytokines involved in host defense. The capacity of VacA to induce a local immune suppression may explain the chronicity of *H. pylori* infections (Gebert et al., 2003). CDT of *A. actinomycetemcomitans*, which induces cell cycle arrest of periodontal fibroblasts, oral epithelial cells or T-lymphocytes in the periodontal environment, deregulates the local immune response and facilitates the bacterial invasion (Belibasakis et al., 2005a,b, 2008; Belibasakis and Bostanci, 2014). Furthermore, it was demonstrated that the ACT cyclomodulins produced by *Bordetella* species and *B*. *anthracis* are capable of interfering with the proliferation and the differentiation of T cells, reducing the production of TNF-α and increasing the production of IL-10 in dendritic cells (Vojtova et al., 2006; Rossi Paccani and Baldari, 2011). The continuous production of ACT during an infection triggers mechanisms that compromise the inflammatory response by inhibiting the regeneration of populations of defense cells (Chou et al., 2008; Gray and Hewlett, 2011). Moreover, ACT was recently found to promote the internalization of bacteria in non-phagocytic cells (Martín et al., 2015). It is likely that an ACT-related increase of bacterial internalization is associated with an ACT-induced cell cycle alteration, similar to that of staphylococcal PSMs.

Bacterial toxins may alter the eukaryotic cell cycle in order to improve their capacity to adhere to and to be internalized by target cells. It was demonstrated that CTX bind to the cell surface via glycolipid receptors GM1, whose highest expression takes place during interphase and the G1 phase (Majoul et al., 2002). Consequently, CTX-induced arrest of cells in the G1 phase amplifies the action of CTX (Figure 4).

A number of toxins with the ability to inhibit different stages of protein synthesis in eukaryotic cells are commonly found in nature (Lemaitre and Girardin, 2013). SubAB and Stx are some of them. While SubAB inhibits the production of pro-inflammatory cytokines and chemokines during infection, Stx, which is also involved in the inhibition of protein synthesis, induces an inflammatory response (Wang et al., 2011). Both SubAB and Stx have been reported to cause cell cycle delay as well as apoptosis (Figure 4; Márquez et al., 2014). A cell cycle delay might be beneficial for an intracellular bacterial establishment due to impaired cellular activity. Moreover the induction of apoptosis likely helps to disperse bacteria within the host as a result of the release of bacteria from apoptotic cells, followed by their migration to other host cells (Gao and Kwaik, 2000; Bergan et al., 2012).

Some bacterial effectors, like the CDT family of toxins produced by a number of unrelated Gram-negative species, lead to cell cycle arrest through their ability to induce chromatin injury. After DNA damage, CDT activates the DNA repair response partly through the ATM kinase that modulates the activation of cell cycle checkpoints, leading to cell type-dependent cell cycle arrest (Arbibe, 2008). The resulting cell cycle arrest alters epithelial cell turnover and thus promotes bacterial colonization. Additionally, bacteria-induced long-lasting DNA damage can result in genetic instability and prone the development of cancer.

Epigenetic modifications that may arise from bacteria-induced injury of chromatin, which maintains the stability and the accessibility of the host genome to the transcriptional machinery (Arbibe, 2008; Bierne and Cossart, 2012), are the focal point of an emerging topic in the field of host-pathogen interactions. There is only limited knowledge as of yet about microbial factors that induce epigenetic modifications which make it possible to impose a pathogen transcriptional signature onto host cells and shape the host immune response (Arbibe, 2008). Consequently, since cyclomodulins are able to induce DNA damage and cell cycle arrest, the following intriguing question is raised: are bacterial cyclomodulins able to promote epigenetic alterations in host cells?

## Concluding remarks

Bacterial cyclomodulins are a growing and heterogeneous family of virulence factors that not only alter host cell cycle progression, but that also interfere with typical host cell activity, including cell differentiation and development that slow down cell and tissue renewal. These actions should be further investigated in *in vivo* models and they might favor host colonization and allow the bacteria to hijack host cell protective functions for their own benefit. Thus, an alteration of the eukaryotic cell cycle might enhance the infective efficiency of bacterial pathogens. It should also be emphasized that bacteria-induced long-lasting DNA damage may induce genetic instability and lead to the development of cancer. Cyclomodulin structures and the mechanisms of bacteria-induced cell cycle alterations dramatically vary depending on the type of bacteria. Most cyclomodulins consist of proteins, but the presence of non-proteinaceous effectors highlight the capacity of bacteria to develop a wide range of toxins with distinct molecular compositions, but with similar actions leading to the enhancement of their fitness during infection. The study of cyclomodulins is an emerging field of research and additional modulins might be identified in a near future. Deciphering the molecular mechanisms by which cyclomodulins hijack the main host cell function, the cell cycle progression, and decoding consequences of such an alteration will ultimately provide us with clues for better understanding the fundamental stages of the host-pathogen interaction, as well as with new targets for the development of therapeutic approaches of bacterial infections.

## Author contributions

RF, AN, and TC wrote the manuscript. AN, YL, MD, and NB composed the figure and created the table. YL, FT, PG, and GL discussed the data and contributed to the writing of the manuscript. VA commented on the manuscript. NB conceived, directed, and contributed to the writing of the manuscript.

### Conflict of interest statement

The authors declare that the research was conducted in the absence of any commercial or financial relationships that could be construed as a potential conflict of interest. The reviewer TF and handling Editor declared their shared affiliation, and the handling Editor states that the process nevertheless met the standards of a fair and objective review.
