# Corrigendum: Heterogeneous Family of Cyclomodulins: Smart Weapons That Allow Bacteria to Hijack the Eukaryotic Cell Cycle and Promote Infections

**DOI:** 10.3389/fcimb.2017.00364

**Published:** 2017-08-14

**Authors:** Rachid A. El-Aouar Filho, Aurélie Nicolas, Thiago L. De Paula Castro, Martine Deplanche, Vasco A. De Carvalho Azevedo, Pierre L. Goossens, Frédéric Taieb, Gerard Lina, Yves Le Loir, Nadia Berkova

**Affiliations:** ^1^STLO, Agrocampus Ouest Rennes, Institut National de la Recherche Agronomique Rennes, France; ^2^Departamento de Biologia Geral, Laboratório de Genética Celular e Molecular, Instituto de Ciências Biológicas, Universidade Federal de Minas Gerais Belo Horizonte, Brazil; ^3^HistoPathologie et Modèles Animaux/Pathogénie des Toxi-Infections Bactériennes, Institut Pasteur Paris, France; ^4^CHU Purpan USC INRA 1360-CPTP, U1043 Institut National de la Santé et de la Recherche Médicale, Pathogénie Moléculaire et Cellulaire des Infections à Escherichia coli Toulouse, France; ^5^International Center for Infectiology Research Lyon, France; ^6^Centre National de la Recherche Scientifique, UMR5308, Institut National de la Santé et de la Recherche Médicale U1111, Ecole Normale Supérieure de Lyon, Université Lyon 1 Lyon, France; ^7^Département de Biologie, Institut des Agents Infectieux, Hospices Civils de Lyon Lyon, France

**Keywords:** eukaryotic cell cycle alteration, bacterial toxins, cyclomodulins, colonization, invasion, infective efficiency, reduced host response

In the original article, there was a mistake in the legend for Figure 3 as published.

It was written: Adenylate cyclase toxin (ACT) binds to an unknown receptor at the cell surface through the pentameric subunit (purple), and the catalytic subunit (brown) is translocated to the cytosol.

The correct legend appears below.

ACT is translocated into the cell cytosol either via binding to the α_m_β_2_ integrin as a cell receptor or by direct translocation to the eukaryotic cells cytosol.

Similarly, there were mistakes in Table 1 as published.

It was indicated

**Table:** 

**Adenylate Cyclase Toxin (ACT)**	AB5 toxin	*B. pertussis*	S1 enzymatic A subunit S2 to S5 binding B subunits	A subunit: acetyltransferase

Enzymatic activity of CNF-1 was indicated as deaminase instead of deamidase.

The corrected Table 1 appears below.

**Table 1:** Cyclomodulins and their key features.

	**Toxin type**	**Species**	**Proteins**	**Enzymatic activity**	**Cell cycle phase delay**
**PROTEIN OR PEPTIDES TOXINS**					
**Cyclomodulins with enzymatic activities**					
Cycle Inhibiting Factor (CIF)	Cysteine protease	*E. coli (EHEC, EPEC)*	2 domains: N-terminal (secretion and	Deamidase	G1/S
			translocation) C-terminal (enzymatic)		G2/M
		*Y. pseudotuberculosis*			
		*Pseudomonas* sp.			
		*Enterobacter* sp.			
		*Serratia* sp.			
γ-glutamyl transpeptidase (GGT)	Enzyme	*H. pylori*	1 protein with 2 chains cleaved by autocatalysis	Gamma-glutamyltransferase	G1/S
Cytolethal Distending Toxin (CDT)	Three globular subunits	*E. col*	CdtB catalytic subunit CdtA and CdtC binding	CdtB subunit: DNase and	G1/S
			subunits	phosphatase	G2/M
		*H. hepaticus*			
		*S. enterica serovar Typhimurium*			
Shiga toxin (Stx) (Verotoxin)	AB5 toxin	*S. dysenteriae E. coli (STEC)*	stxA enzymatic subunit StxB binding subunit	A subunit: N-glycosidase	S
Subtilase AB (SubAB)	AB5 toxin	*E. coli (STEC)*	SubA enzymatic subunit SubB binding subunit	A subunit: protease	G1/S
Anthrax toxin (Edema toxin / Lethal toxin)	Tripartite toxin	*B. anthracis*	Edema and/or Lethal factor (A enzymatic subunit) Protective Antigen (B binding subunit)	Edema factor: adenylate cyclase Lethal factor: zinc metalloprotease	G1/S
Cholera toxin (Ctx)	AB5 toxin Oligomeric complex	*V. cholerae*	CTA (enzymatic subunit) comprises CTA1 and CTA2 domains CTB (B binding subunit)	ADP-ribosyltransferase	G1/S
Adenylate Cyclase Toxin (ACT)	RTX family of toxin	*B. pertussis*	2 domains: N- terminal (enzymatic) C-terminal (pore-forming)	Adenylate cyclase	G1/S
Vacuolating cytotoxin (VacA)	Pore-forming toxin	*H. pylori*	3 domains (p33, p55, β-barrel)	Hypothetically	G1/S
Cytotoxic Necrotizing Factor 1 (CNF1)	Non canonical AB toxin	*E. coli*	3 domains: N-terminal (binding) C-terminal (enzymatic) Central (translocation)	Deamidase	G2/M
**Cyclomodulins without enzymatic activities**					
Panton–Valentine leukocidin (PVL)	β-pore-forming toxin Bi-component toxin	*S. aureus*	LukS-PV LukF-PV	No	G0/G1
Phenol soluble modulins (PSMs)	Peptides	*S. aureus*	PSMα, PSMβ, PSMγ	No	G2/M
**NON-PROTEINACEOUS CYCLOMODULINS**					
Mycolactone	Macrolide	*M. ulcerans*	–	No	G0/G1

Finally, it was written that “Similar to *B. anthracis, B. pertussis* produces an adenylate cyclase toxin (ACT), which belongs to the AB5 toxin family (Figure 3) (Melvin et al., 2014).”

A correction has been made to section Cyclomodulins: Protein Toxins or Peptide Toxins, subsection Cyclomodulins with Enzymatic Activities, sub-subsection Adenylate cyclase toxin, first paragraph. The corrected paragraph appears below:

*Bordetella pertussis*, a Gram-negative bacterial pathogen, is responsible for respiratory infections manifested by whooping cough, with possible lethal complications (Table 1).

Similar to *B. anthracis, B. pertussis* produces an adenylate cyclase toxin (ACT) (Figure 3) (Melvin et al., 2014). ACT of *B. pertussis* is a ~200 kDa protein consisting of two functional domains: an N- terminal adenylate cyclase enzyme domain (AC domain) and a pore-forming or hemolysin domain (Hly domain), which belongs to the RTX (Repeats in Toxin) family (Carbonetti, 2010). ACT displays the hemolytic/pore-forming activity along with the adenylate cyclase enzymatic activity (Basler et al., 2006). ACT is released by the Type I bacterial secretion system (Glaser et al., 1988). The Hly domain is required for the delivery of the AC domain into the cell cytosol either via binding to the α_m_β_2_ integrin (CD11b/CD18) as a cell receptor or by direct translocation to the eukaryotic cells cytosol (Guermonprez et al., 2001; Eby et al., 2010).

The authors apologize for this error and state that this does not change the scientific conclusions of the article in any way.

## Conflict of interest statement

The authors declare that the research was conducted in the absence of any commercial or financial relationships that could be construed as a potential conflict of interest.
